# A model based on Chinese thyroid imaging reporting and data systems for predicting Bethesda III/IV thyroid nodules

**DOI:** 10.3389/fendo.2025.1442575

**Published:** 2025-03-03

**Authors:** An Wei, Yu-Long Tang, Shi-Chu Tang, Xin-Wu Cui, Chao-Xue Zhang

**Affiliations:** ^1^ Department of Ultrasound, Hunan Provincial People’s Hospital/The First Affiliated Hospital of Hunan Normal University, Changsha, Hunan, China; ^2^ Department of Ultrasound, The First Affiliated Hospital of Anhui Medical University, Hefei, Anhui, China; ^3^ Department of Thyroid Surgery, Hunan Cancer Hospital/The Affiliated Cancer Hospital of Xiangya School of Medicine, Central South University, Changsha, Hunan, China; ^4^ Department of Medical Ultrasound, Hunan Cancer Hospital/The Affiliated Cancer Hospital of Xiangya School of Medicine, Central South University, Changsha, Hunan, China; ^5^ Department of Medical Ultrasound, Tongji Hospital, Tongji Medical College, Huazhong University of Science and Technology, Wuhan, Hubei, China

**Keywords:** ultrasound, thyroid nodule, the Bethesda system for reporting thyroid cytology, Chinese thyroid imaging reporting and data systems, fine needle aspiration

## Abstract

**Objectives:**

This study aimed to explore the performance of a model based on Chinese Thyroid Imaging Reporting and Data Systems (C-TIRADS), clinical characteristics, and other ultrasound characteristics for the prediction of Bethesda III/IV thyroid nodules before fine needle aspiration (FNA).

**Materials and methods:**

A total of 855 thyroid nodules from 810 patients were included. All nodules underwent ultrasound examination before FNA. All nodules were categorized according to the C-TIRADS criteria and classified into two groups, Bethesda III/IV and non-III/IV thyroid nodules, using cytologic diagnosis as the gold standard. The clinical and ultrasonographic characteristics of the nodules in the two groups were compared, and independent predictors of Bethesda III/IV nodules were determined by univariate and multivariate logistic regression analyses, based on which a prediction model was constructed. The predictive efficacy of the model was compared with that of C-TIRADS alone by sensitivity, specificity, and area under the curve (AUC).

**Results:**

Our study found that the C-TIRADS category, homogeneous echotexture, blood flow signal present, and posterior echo unchanged were independent predictors for Bethesda III/IV thyroid nodules. Based on multiple logistic regression, a predictive model was established: Logit (p)= - 4.213 + 0.965 × homogeneous echotexture+ 1.050 × blood flow signal present + 0.473 × posterior echo unchanged+ 2.859 × C-TIRADS 3 + 2.804 × C-TIRADS 4A + 1.824 × C-TIRADS 4B + 0.919 × C-TIRADS 4C. The AUC of the model among all nodules was 0.746 (95%CI: 0.710-0.782), 0.779 (95%CI: 0.730-0.829) among nodules with a diameter (D) > 10mm, and 0.718 (95%CI: 0.667-0.769) among nodules with D ≤ 10mm, which were significantly higher than that of the C-TIRADS alone.

**Conclusion:**

We developed a predictive model for Bethesda III/IV thyroid nodules that is better for nodules with D > 10mm FNA operators can choose the optimal puncture strategy based on the prediction results to improve the rate of definitive diagnosis of the first FNA of Bethesda III/IV nodules and thus reduce repeat FNA.

## 1 Introduction

The prevalence of thyroid nodules has been steadily increasing over the years, currently up to 68% (1, 2). Among them, 7-15% are malignant nodules (1–3). Precise identification and standardized management of these malignant nodules have always been a clinical imperative. High-resolution ultrasound serves as the gold standard for assessing thyroid nodule size due to its advantages in providing clear and real-time imaging. It is also the primary choice for clinical screening of thyroid nodules (1, 4). Ultrasound exhibits a specificity exceeding 90% in differentiating between benign and malignant thyroid nodules; however, its sensitivity is slightly lower at only 67-82% (5).

To standardize thyroid ultrasound reporting and strengthen effective communication between physicians, Horvath first launched the Thyroid Imaging Reporting and Data System (TIRADS) in 2009 to evaluate the malignant risk of thyroid nodules. Subsequently, many international associations, including the American College of Radiology (ACR) and the American Thyroid Association (ATA), issued guidelines for the diagnosis of thyroid nodules (6–11). In 2020, the Chinese Medical Association also launched the Chinese Thyroid Imaging Reporting and Data System (C-TIRADS) suitable for Chinese people (12).

When malignant nodules are suspected on ultrasound, fine needle aspiration (FNA) is recommended by all TIRADS to further clarify the diagnosis. To standardize terminology and facilitate communication, the National Cancer Institute (NCI) established the Bethesda Thyroid Cytopathology Reporting System (TBRSTC) in 2010 (13). This system categorizes FNA findings into six categories: Bethesda I to Bethesda VI. Of these, Bethesda III and IV nodules are referred to as indeterminate nodules because there is insufficient cytologic evidence to support a diagnosis of benign or malignant.

Because the malignancy rate of Bethesda III/IV nodules is as high as 34% to 52%, repeat FNA combined with molecular or genetic sequencing or even diagnostic lateral lobectomy is required for a definitive diagnosis (13–18). Patients not only have to pay higher medical expenses but also have to endure the physical and psychological trauma caused by repeated FNA before obtaining a definitive diagnosis. If we can predict Bethesda III/IV nodules before the first FNA, we can optimize the FNA strategy in advance to improve the diagnosis rate of the first FNA and avoid repeat punctures. Currently, researchers are actively exploring the differentiation of benign and malignant Bethesda III/IV thyroid nodules by ultrasound techniques (19). However, there are few studies on the prediction of Bethesda III/IV thyroid nodules by ultrasound, and there are no reports on the prediction of Bethesda III/IV thyroid nodules based on C-TIRADS.

This study aimed to investigate the possibility of predicting Bethesda III/IV thyroid nodules by clinical features, conventional ultrasound, and C-TIRADS classification and to construct a prediction model that would facilitate operators to predict whether a nodule is a Bethesda III/IV category nodule before the first FNA.

## 2 Materials and methods

### 2.1 Study objects

This study was approved by the Medical Ethics Committee of our hospital for waiver of informed consent(2023 No.: LY-2023-89). Patients with thyroid nodules who underwent ultrasound examination and FNA in our hospital from January 2021 to November 2023 were selected. The inclusion criteria were as follows: (1) age ≥18 years old; (2) Solid or predominantly solid nodules (> 75%); (3) FNA was performed within 1 month after ultrasound examination and classified according to criteria of TBSRTC. (4) There was no acute or subacute thyroiditis associated with ultrasound examination. (5) no previous history of thyroid puncture, surgery, or ablation; Exclusion criteria:(1) ultrasound image quality could not meet the requirements; (2) FNA nodules without matched ultrasound images.

### 2.2 Apparatus and methods

The patient’s thyroid region was explored by the same ultrasound expert with 10 years of experience in thyroid ultrasound using a supersonic Aixplorer system (Super Sonic Imagine, Aix en Provence, France) with an L15-4 linear array transducer. When performing a color Doppler ultrasound examination, the scale was adjusted to ≤ 10cm/s, and the probe was gently placed to avoid affecting the blood flow imaging (12, 20). Clear and complete ultrasound images of thyroid nodules were saved as JPEG files. The location of the nodules (right/left lobe, isthmus, upper and lower parts, inner and outer parts, deep and superficial parts) and the maximum diameter of the nodules were recorded. The patient’s age, gender, and other data were collected through the Picture Archiving and Communication System (PACS) and medical record system. According to the cytological results after FNA, the nodules were divided into the Bethesda III/IV group and the non-III/IV group.

### 2.3 C-TIRADS categorizing

All nodules were categorized by two ultrasound experts according to the categorization methods in the C-TIRADS guidelines (see **Supplementary Material**) (12). If the two experts did not agree on the classification of the nodule, consensus was reached in consultation with a third ultrasound expert. Ultrasound features not included in the C-TIRADS guidelines were analyzed separately. Fifty thyroid nodules were randomly selected, and the consistency of the two experts in the classification of thyroid nodules was compared. After a week, the 50 nodules were classified again by one of the experts, and then the consistency of the two classifications by the expert was compared.

### 2.4 Statistical methods

SPSS 26.0 was adopted for all statistical analyses. Shapiro-Wilk test was used to verify the normal distribution of data. Measurement data were expressed as mean ± standard deviation (X ± s) and analyzed by independent sample t-test or non-parametric test. The count data of clinical characteristics, ultrasound characteristics, and C-TIRADS category of the two groups were compared using X2 test or Fisher’s exact test. The threshold of statistical significance was *P*<0.05. The statistically significant variables in the univariate analysis were added to the multivariate logistic regression analysis to determine independent predictors of Bethesda III/IV thyroid nodules. The receiver operating characteristic (ROC) was used to calculate the predictive performance of the independent predictors and the predictive model, and the sensitivity, specificity, and area under the curve (AUC) of the independent predictors and the prediction model were compared. Intra-class correlation coefficient (ICC) was used to evaluate the inter-observer and intra-observer agreement.

## 3 Results

### 3.1 The general characteristics of the nodules and the patients

After strict screening according to the inclusion and exclusion criteria, a total of 855 thyroid nodules in 810 patients (765 patients with 1 nodule and 45 patients with 2 nodules) were included in this study. There were 181 males (186 nodules) and 635 females (699 nodules), aged from 18 to 77 years, with an average age of 43.9 ± 12.6 years. Among the 855 nodules, there were 224 (26.2%) Bethesda III/IV nodules, including 126 (14.7%) Bethesda III and 98 (11.5%) Bethesda IV nodules; there were 631 (73.8%) non-Bethesda III/IV nodules. As shown in **Table 1**, Of these 810 patients, 629 (77.7%) were female and 181 (22.3%) were male. There was no significant difference in age and gender distribution between the two groups (*P* > 0.05).

**Table 1:** Number, size of nodules and age of patients.

Parameter	The number of nodules	patient	Bethesda		*P*
			III/IV	Non III/IV	
All nodules	855	810	224	631	
Age		43.86±12.56	43 .81±12.87	43 .89±12.42	0.836
Female	669	629	175	494	0.959
Male	186	181	49	137	
D ≤ 10mm	466	436	119	347	
Age		43.85±11.38	44.46±12.15	43.64±11.11	0.611
Female	377	351	95	282	0.731
Male	89	85	24	65	
D > 10mm	389	374	105	284	
Age		43.89±13.81	43.08±13.67	44.20±13.87	0.453
Female	292	278	80	212	0.755
Male	97	96	25	72	

*P-value<0.05 was considered statistically significant.

D, diameter.

The maximum diameter of nodules ranged from 2 to 56 mm, with an average of 12.59 ± 8.73mm. Among the nodules with max diameter (D) ≤ 10 mm, there were 13 C-TIRADS category 3 and 171 C-TIRADS category 4A nodules. All C-TIRADS category 3 nodules underwent FNA due to the patient’s request for a definitive diagnosis, including 1 Bethesda V nodule, 5 Bethesda IV nodules, 1 Bethesda III nodule, 4 Bethesda II nodules, and 2 Bethesda I nodules. Only 1 nodule was surgically resected and pathologically diagnosed as a low-grade malignant. There were 14 (8.1%) Bethesda VI and 36 (21%) Bethesda V nodules in these 171 C-TIRADS category 4A nodules.

### 3.2 The ultrasound characteristics of the nodules

The ultrasound characteristics of the two groups of nodules are summarized in **Table 2**. There were significant differences between the two groups in hypoechogenicity, microcalcifications, regular margin, extrathyroidal extension, taller-than-wide regular shape, homogeneous echotexture, blood flow signal present, posterior echo enhancement, or unchanged (*P* < 0.05). There was no significant difference in the specific location of nodules between the two groups (*P* > 0.05). C-TIRADS criteria included taller-than-wide shape, microcalcifications, ill-defined or irregular margins, and extrathyroidal extension. hypoechogenicity, regular shape, homogeneous echotexture, blood flow signal present, posterior echo enhancement or unchanged, which were statistically significant differences between groups, were not included in the C-TIRADS criteria and were therefore included in the regression analysis along with the C-TIRADS classification.

**Table 2:** The ultrasound characteristics of thyroid nodules.

Characteristics		Bethesda III/IV	Non Bethesda III/IV	X2/Z/t
		(n=224)	(n=631)	
Echogenicity				
hypoechogenicity	Y ( n=740 )	185 ( 82.6% )	555 (88.0% )	4.090
	N ( n=115)	39( 17.4% )	76 ( 12.0 % )	
Markedly hypoechogenicity	Y ( n=46 )	9 (4.0% )	37 ( 5.9% )	1.106
	N (n=809 )	215 (96.0% )	594 ( 94.1% )	
Hyperechoic	Y ( n= 11 )	5 ( 2.2 % )	6 ( 1.0 % )	2.137
	N ( n= 844 )	219 ( 97.8 % )	625 ( 99.0 % )	
Isoechoic	Y (n=29)	9(4.0%)	20(3.2%)	0.363
	N(n=826)	215(96.0%)	611(96.8%)	
Echogenic foci				
Microcalcifications	Y ( n=420)	81 ( 36.2% )	339 (53.7% )	20.405
	N ( n=435)	143 ( 63.8% )	292( 46.3% )	
Margin				
Irregular margin	Y ( n=637 )	153( 68.3 % )	484 ( 76.70% )	6.141
	N ( n=218 )	71 ( 31.7 % )	147 (23.3% )	
Ill-defined margin	Y ( n= 618 )	139 ( 62.1 % )	479 ( 75.9 % )	0.237
	N ( n= 237 )	85 ( 37.9 % )	152 ( 24.1 % )	
Extrathyroidal extension	Y ( n=89)	2( 1.0% )	87 ( 16.0% )	29.910
	N ( n=646)	190 ( 99.0% )	456 ( 84.0% )	
taller-than-wide	Y ( n=376 )	81 ( 36.2% )	295 (46.8% )	7.526
	N ( n=479 )	143 ( 63.8% )	336 ( 53.2% )	
Regular form	Y ( n=245 )	97 ( 43.3% )	148 (23.5% )	31.858
	N ( n=610 )	127 ( 56..7% )	483 ( 76.5 % )	
Homogeneous	Y ( n=53 )	25 ( 11.2% )	28 ( 4.4% )	12.852
	N ( n=802 )	199 ( 88.8% )	603 (95.6% )	
Blood flow signal present	Y( n=690)	199 ( 88.8% )	491 (77.8% )	12.905
	N( n=165)	25 ( 11.2% )	140 ( 22.2% )	
Posterior features				
Enhancement	Y ( n=61)	26( 11.6 % )	35 ( 5.5% )	9.164
	N ( n=794)	198 ( 88.4 % )	596 (94.5% )	
unchanged	Y ( n=617)	148(66.1%)	469(74.3%)	5.608
	N ( n=238 )	76 ( 33.9% )	162 ( 25.7% )	
Shadowing	Y ( n=177 )	50 ( 22.3% )	127 ( 20.1% )	0.485
	N ( n=678)	174 ( 77.7% )	504 ( 79.9% )	
Location				
Inner	Y ( n= 40 )	6 ( 2.7 % )	34 ( 5.4 % )	2.722
	N ( n= 815 )	218 ( 97.3 % )	597 ( 94.6 % )	
Outer	Y ( n= 58 )	12 ( 5.4 % )	46 ( 7.3 % )	0.977
	N ( n= 797 )	212 ( 94.6 % )	585 ( 92.7 % )	
Deep	Y ( n=193)	53( 23.7% )	140 ( 22.2% )	0.205
	N ( n=662)	171 ( 76.3 % )	491 (77.8% )	
Superficial	Y ( n=254 )	56 ( 25.0% )	198 ( 31.4% )	3.221
	N ( n=601)	168 ( 75.0% )	433 (68.6% )	
Upper	Y ( n=118)	29( 12.9 % )	89 ( 14.1% )	0.186
	N ( n=737)	195 ( 87.1 % )	542 (85.9% )	
Lower	Y ( n=192)	47( 21.0 % )	145 ( 23.0% )	0.379
	N ( n=663)	177 ( 79.0 % )	486 (77.0% )	
Position				
Left lobe	( n=392 )	101 ( 45.1 % )	291 (46.1 % )	0.080
Right lobe	( n=432 )	115 ( 51.3 % )	317 ( 50.2 % )	
Isthmus	( n=31 )	8 ( 3.6 % )	23 ( 3.6 % )	
Maximum diameter		10 ( 6.8~17.0 )	10 ( 7.0~15.0 )	-0.170

Patient's age is expressed as mean ± standard deviation.

*P-value<0.05 was considered statistically significant.

### 3.3 The C-TIRADS categories of the nodules

There was a significant difference in the composition of C-TIRADS categories between the two groups of nodules (*P* < 0.001), as shown in **Table 3**. Of the 224 Bethesda III/IV nodules, 25 (11.2%) were in C-TIRADS category 3, and 126 (56.3%) were in C-TIRADS category 4A, which was significantly higher than that of non-Bethesda III/IV nodules (4.8%, *P* = 0.001, 28.7%, *P* < 0.001). Of the 631 non-Bethesda III/IV nodes, 200 (31.7%) were in C-TIRADS category 4B, 138 (21.9%) were in C-TIRADS category 4C, and 82 (13%) were in C-TIRADS category 5, which were significantly higher than the non-Bethesda III/IV nodes (23.2%, 7.6%, 1.8%, P <0.001). ROC curves based on C-TIRADS categories showed that the best threshold for distinguishing the two groups of nodules was C-TIRADS 4B. The inter-observer ICC for the classification of thyroid nodules by two experts was 0.877, and the intra-observer ICC was 0.962.

**Table 3:** C-TIRADS category of the two groups of nodules.

C-TIRADS category	Score	Bethesda III-IV		The total number of nodules	*P*
		Yes	Non		
1	–	–	–	–	–
2	-1	–	–	–	–
3	0	25 ( 11.2% )	30 ( 4.8% )	55 ( 6.4% )	0.001*
4A	1	126 ( 56.3% )	181 ( 28.7% )	307 ( 35.7% )	<0.001*
4B	2	52 ( 23.2% )	200 ( 31.7% )	252 ( 29.5% )	0.017*
4C	3-4	17 ( 7.6% )	138 ( 21.9% )	155 ( 18.1% )	<0.001*
5	5	4 (1.8% )	82 ( 13.0% )	86 ( 10.1 %)	<0.001*
The total number of nodules	–	224 ( 100% )	631 ( 100% )	855 ( 100% )	<0.001*

*P-value<0.05 was considered statistically significant.

C-TIRADS, the Chinese Thyroid Imaging Reporting and Data System.

### 3.4 The predictors of Bethesda III/IV thyroid nodules

**Figure 1:**
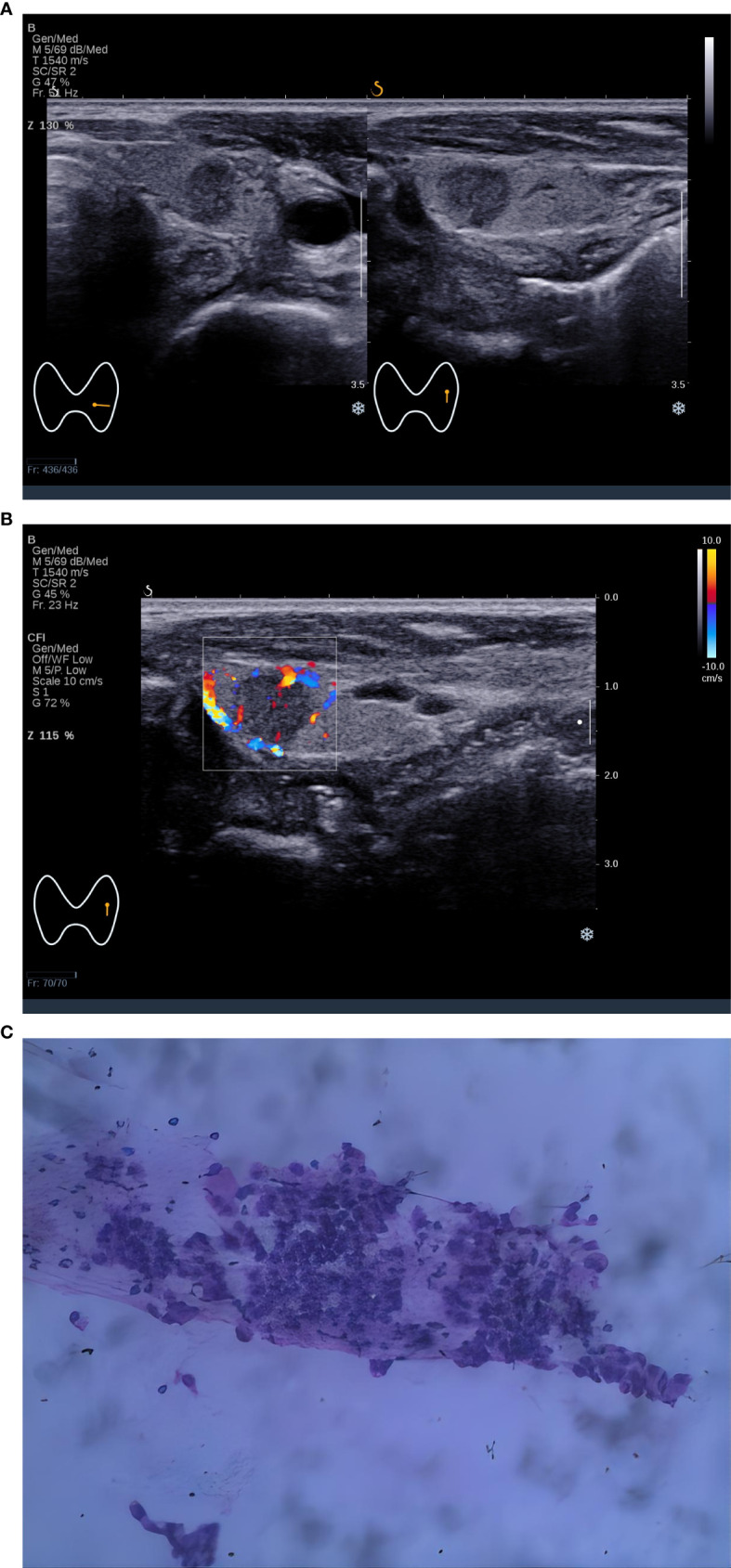
A 5×5 mm thyroid nodule in the left lobe of a 44-year-old woman. **(A)** Two-dimensional ultrasound of transverse and longitudinal sections showed a nodule with homogeneous echotexture, regular shape, posterior echo unchanged, and the C-TIRADS category was 4A, **(B)** Color Doppler ultrasound showed blood flow in the nodule. The predictive value calculated by the logistic regression formula was 0.4114 (> 0.2946), which was considered to be Bethesda III/IV. **(C)** Cytological pathological examination showed Bethesda III.

The binary logistic regression analysis of the C-TIRADS category and other ultrasound characteristics of the nodules of the two groups are summarized in **Table 4**. Regular shape, hypoechogenicity, and posterior echo enhancement were excluded because *P* > 0.05. C-TIRADS category, homogeneous echotexture, blood flow signal present, and posterior echo unchanged were independent predictors for Bethesda III/IV thyroid nodules. The predictive model was established based on backward stepwise binary logistic regression analysis: Logit (p)= - 4.213 + 0.965 × homogeneous echotexture + 1.050 × blood flow signal present + 0.473 × posterior echo unchanged+ 2.859 × C-TIRADS 3 + 2.804 × C-TIRADS 4A + 1.824 × C-TIRADS 4B + 0.919 × C-TIRADS 4C, as show in **Figure 1**.

**Table 4:** Multivariate logistic regression analysis of clinical data, conventional ultrasound, and C-TIRADS.

	B	*P*	OR	95% C.I for OR	
Regular form	0.247	0.196	1.280	0.880	1.860
Hypoechogenicity	0.232	0.357	1.261	0.770	2.065
Homogeneous echotexture	0.965	0.003	2.625	1.394	4.945
Blood flow signal present	1.050	0.000	2.859	1.771	4.614
Posterior echo unchanged	0.473	0.010	1.604	1.119	2.299
Posterior echo enhancement	0.073	0.834	1.075	0.5468	2.118
C-TIRADS category		0.000			
3	2.859	0.000	17.452	5.517	55.208
4A	2.804	0.000	16.517	5.857	46.576
4B	1.824	0.001	6.199	2.156	17.817
4C	0.919	0.111	2.506	0.810	7.751
Constant	-4.213	0.000	2.625	1.394	4.945

*P-value<0.05 was considered statistically significant.

C-TITADS: the Chinese Thyroid Imaging Reporting and Data System.

### 3.5 Comparing the predictive performance of C-TIRADS alone and the model for Bethesda III/IV nodules

**Figure 2:**
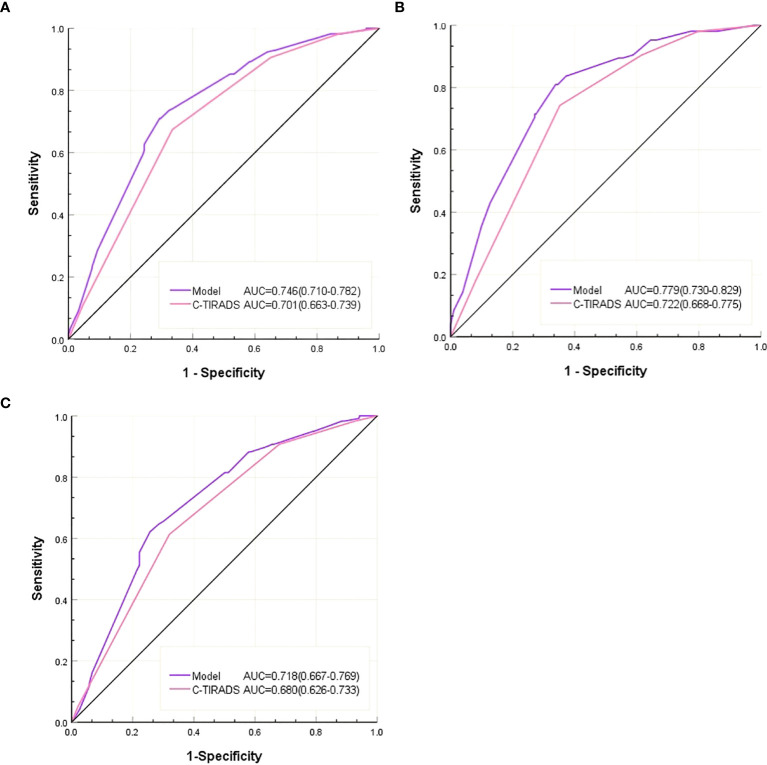
**(A)** ROC of the C-TIRADS alone and the predictive model in all nodules, **(B)** ROC of the C-TIRADS alone and the predictive model in the D ≤ 10mm nodules. **(C)** ROC of C-TIRADS alone and predictive model for D > 10mm nodules.

As shown in **Table 5**, the AUC of the predictive model was 0.746 (95% CI: 0.710-0.782), which was significantly higher than that using C-TIRADS alone (AUC=0.701, *P* =0.014) (**Table 5**, **Figure 2A**). Compared with using C-TIRADS alone. The sensitivity and specificity of the predictive model were 71.0% and 70.7%, respectively.

**Table 5:** The AUC of C-TIRADS and predictive model.

	AUC	*P*	The sensitivity (%)	*P*	The specificity (%)	*P*
all nodules						
C-TIRADS	0.701 (0.663-0.739)	0.014	67.4 (61.2-73.6)	0.415	66.6 (62.9-70.3)	0.115
The model	0.746 (0.710-0.782)		71.0 (65.0-77.0)		70.7 (67.1-74.2)	
D≤10mm						
C-TIRADS	0.680 (0.626-0.733)	0.047	61.3 (52.5-70.2)	0.894	68.0 (63.1-72.9)	0.065
The model	0.718 (0.667-0.769)		62.2 (53.3-71.0)		74.4 (69.7-79.0)	
D>10mm						
C-TIRADS	0.722 (0.668-0.775)	<0.001	74.3 (65.8-82.8)	0.246	64.8 (59.2-70.4)	0.724
The model	0.779 (0.730-0.829)		81.1 (73.3-88.6)		66.2 (60.7-71.7)	

*P-value<0.05 was considered statistically significant.

AUC, area under the curve.

TITADS, the Chinese Thyroid Imaging Reporting and Data System.

D, diameter.

In nodules with D ≤ 10 mm, the AUC of the predictive model (0.718) was higher than that of C-TIRADS alone (0.680, *P* = 0.047) (**Table 5**, **Figure 2B**). The sensitivity and specificity of predictions using C-TIRADS alone were 61.3% and 68.0%, respectively. The sensitivity and specificity of model prediction were 62.2% and 74.4%, respectively. The difference was not statistically significant, but the difference in specificity was close to 0.05 (*P* = 0.065).

In nodules with D > 10 mm, the AUC of the model predicting Bethesda III/IV nodules (0.779) was higher than that of C-TIRADS alone (0.722, *P <* 0.001) (**Table 5**, **Figure 2C**). The sensitivity and specificity of C-TIRADS alone were 74.3% and 64.8%, respectively. Those of the model were 81.1% and 66.2%, respectively. The prediction sensitivity of the model in nodules with D > 10 mm was higher than those in nodules with D ≤10mm (*P* = 0.002). The prediction specificity of the model in nodules with D ≤10mm was higher than those in nodules with D > 10 mm (*P* = 0.025).

DeLong’s test showed that the model’s AUC was significantly higher than the AUC of C-TIRADS alone (P < 0.05) in all nodules, in nodules with D > 10 mm or nodules with D ≤10mm. The AUC of the model increased from 0.722 with C-TIRADS alone to 0.776 in nodules with D > 10 mm and from 0.680 to 0.712 in nodules with D ≤10mm. The increase in AUC of the model was more significant in nodules with D > 10 mm.

## 4 Discussion

In this study, we compared the clinical and ultrasound characteristics of Bethesda III/IV thyroid nodules with those of non-III/IV thyroid nodules. The findings showed that the C-TIRADS category, blood flow signal present, homogeneous echotexture, and posterior echo unchanged were independent predictors of Bethesda III/IV thyroid nodules. The predictive model based on the C-TIRADS category and other ultrasound characteristics predicted Bethesda III/IV thyroid nodules with an AUC of 0.746. The predictive sensitivity of the model for Bethesda III/IV nodules with D > 10 mm was 81.1%, with an AUC of 0.779, which was better than that for Bethesda III/IV nodules with D ≤ 10 mm. Based on the predictive results of the model, clinicians could optimize the puncture strategy before FNA by a variety of methods, such as combining molecular or genetic testing, changing experienced operators, conducting on-site evaluation by a cytopathologist, or even coarse-needle histological biopsy, to improve the definitive diagnostic rate of the first puncture while avoiding physical and psychological injuries as well as increased economic and time costs for the patient caused by repeated FNA (13, 21, 22). The precise optimization of the puncture strategy also avoids the waste of medical resources.

Individual ultrasound features are subjectively affected by observers, while comprehensive analysis using TIRADS could improve inter-observer consistency (23). Inspired by the Breast Imaging Reporting and Data System (BI-RADS) guidelines, TIRADS has been released in several versions since it was first proposed in 2009, including C-TIRADS (11, 12, 24). In a study of 1096 thyroid nodules with histopathological results, researchers compared five TIRADS, including C-TIRADS, and found that C-TIRADS exhibited the highest specificity (82.3% vs. 70.5%, 62.0%, 55.4%, 66.7%, *P*<0.05), the lowest rate of unnecessary biopsies (49.02% vs. 50.25%, 55.99%, 53.09%, 58.39%, *P*<0.001), the highest accuracy (76.0% vs. 72.5%, 71.8%, 67.8%, 72.5%, *P*<0.05), the highest AUC (0.816 vs. 0.789, 0.773, 0.763, 0.734, *P* < 0.05) (25). Therefore, we chose C-TIRADS for the overall evaluation of thyroid nodules. We observed a lower incidence of Bethesda III/IV nodules among C-TIRADS category 4C and 5 nodules, which may be due to the higher positive predictive value of C-TIRADS for malignant nodules (25). Bethesda III/IV nodules were 17 and 6 times more common in C-TIRADS categories 3-4A and 4B, respectively, than in category 5 nodules, whereas there was no significant difference in C-TIRADS categories 4C and 5. This suggests that Bethesda III/IV nodules occur predominantly in the lower C-TIRADS categories. Therefore, when deciding to perform FNA on C-TIRADS category 3-4B nodules, it is possible to predict in advance whether it is a Bethesda III/IV nodule and selectively optimize the FNA strategy based on the prediction results to avoid repeated FNA. As for the specific optimization strategy, clinicians need to make reasonable choices according to the specific conditions of patients. For example, core needle biopsy is not suitable for patients with a high risk of bleeding and poor pain tolerance. Operator switching and on-site evaluation may be a more appropriate option for patients who cannot afford expensive molecular testing.

Women accounted for 77.7% (629/810) and men for 22.3% (181/810) of the patients included in this study, which is consistent with the previously reported male-to-female ratio (17). However, there was no statistically significant difference in the gender composition and age composition between the Bethesda III/IV nodule group and the non-Bethesda III/IV category nodule group.

The two groups of nodules differed in the ultrasound characteristics of hypoechogenicity, regular shape, homogeneous echotexture, blood flow signal present, posterior echo enhancement, or unchanged, which were not included in the C-TIRADS category criteria (12). For a comprehensive assessment, these ultrasound characteristics were included in regression analysis along with the C-TIRADS category of the nodule. Ultimately, the independent predictors of Bethesda III/IV thyroid nodules were the homogeneous echotexture, blood flow signal present, posterior echo unchanged, and the C-TIRADS category. On this basis, we developed a predictive model. The AUC, sensitivity, and accuracy of the model were higher than the prediction using C-TIRADS alone.

The AUC of the prediction model was 0.779 in nodules with D > 10 mm, which was significantly higher than 0.718 in nodules with D ≤ 10mm. The sensitivity of the prediction model in nodules with D > 10 mm (81.1%), was significantly higher than 62.2% in nodules with D ≤10mm (*P* = 0.002). However, the specificity was lower in nodules with D > 10 mm than in nodules with D ≤ 10mm, indicating that the prediction model could predict more than 80% of Bethesda III/IV nodules in nodules with D > 10 mm. There is room for further improvement in the performance of our model. In the future, we plan to expand the sample size, add predictive variables such as contrast-enhanced ultrasound, shear wave elastography, and other multimodal ultrasound imaging data, and incorporate more patient-specific factors such as thyroid serum biochemical indicators such as thyroid stimulating hormone (TSH) to improve the predictive performance of the model and improve its clinical utility. To ensure the consistency of image quality, if the images come from different operators, we recommend that operators receive standardized training and pass the examination before collecting images, and it is best to have many years of operation experience and be familiar with C-TIRADS scoring standards.

The development of artificial intelligence (AI) technology has gradually penetrated the field of medical diagnosis and treatment. Some scholars have developed ThyGPT through ChatGPT, which can effectively communicate with doctors through human-computer interaction, make accurate judgments, and improve the efficiency of diagnosis (26). Yao J et al. used AI technology further to identify the pathological category of BethesdaIV thyroid nodules, and the AUC was 0.90-0.95 (27). Based on our prediction of Bethesda III/IV thyroid nodules, we can further predict the possible pathological classification of nodules by AI, which can further reduce the FNA rate and optimize the diagnostic process of thyroid nodules.

The limitations of this study were as follows. First, this study was a single-center retrospective study that only included patients undergoing FNA, and nodules that did not meet the recommended criteria for FNA were excluded except for some nodules requested by patients, leading to potential selection bias. Second, in this study, only 36 of 126 Bethesda III thyroid nodules obtained postoperative pathological diagnosis. Among them, 28 were papillary carcinomas, 2 were follicular tumors, and 6 were benign nodules. Among the 98 Bethesda IV nodules, only 7 obtained postoperative pathological diagnosis, including three papillary carcinomas, 3 low-grade malignant nodules, and 1 benign nodule. Due to the lack of surgical pathological diagnosis of all nodules in this study, the malignancy rate of Bethesda III/IV nodules could not be further determined. Third, this study did not compare interobserver and intraobserver differences. Finally, external validation to assess the prediction performance of the model has not been performed and should be considered in future studies.

## 5 Conclusion

In conclusion, our study found that homogeneous echotexture, blood flow signal present, unchanged posterior echo, and C-TIRADS category were independent predictors of Bethesda III/IV thyroid nodules. On this basis, a prediction model for Bethesda III/IV thyroid nodules was constructed, which had good predictive efficacy, especially for nodules with D ≥ 10 mm. The model could help clinicians predict Bethesda III/IV nodules based on ultrasound characteristics before FNA, and then optimize the FNA strategy by combining genetic and molecular testing, puncture by skilled FNA operators, and rapid assessment by pathologists on site to improve the definitive diagnosis rate of the first-time FNA and reduce the physical and psychological trauma and increased healthcare costs for patients due to repeat FNA (21, 28–30). Precise optimization can also avoid the waste of medical resources caused by blindly expanding the number of patients.

## Data Availability

The datasets presented in this article are not readily available because the data that support the findings of this study are available from the corresponding author, upon reasonable request. Requests to access the datasets should be directed to weian1976@163.com.
